# The BDNF-FoxO1 Axis in the medial prefrontal cortex modulates depressive-like behaviors induced by chronic unpredictable stress in postpartum female mice

**DOI:** 10.1186/s13041-020-00631-3

**Published:** 2020-06-12

**Authors:** Jing Liu, Fantao Meng, Juanjuan Dai, Min Wu, Wentao Wang, Cuilan Liu, Di Zhao, Hongcai Wang, Jingyan Zhang, Chen Li

**Affiliations:** 1 Institute for Metabolic & Neuropsychiatric Disorders, Binzhou Medical University Hospital, No. 661 Huanghe 2nd Road, Binzhou, 256603 Shandong China; 2 Cancer Research Institute, Binzhou Medical University Hospital, No. 661 Huanghe 2nd Road, Binzhou, 256603 Shandong China; 3 Neurosurgery, Binzhou Medical University Hospital, No. 661 Huanghe 2nd Road, Binzhou, 256603 Shandong China; 4 Department of Neurology, Binzhou Medical University Hospital, No. 661 Huanghe 2nd Road, Binzhou, 256603 Shandong China

**Keywords:** Chronic unpredictable stress, Medial prefrontal cortex, BDNF, FoxO1, Postpartum depression

## Abstract

Postpartum depression (PPD) is a serious psychiatric disorder, affecting not only the childbearing women but also the health of their offsprings. The brain-derived neurotrophic factor (*Bdnf*) gene is an important target gene for the study of depression and antidepressant therapy. FoxO1, belonging to the FoxO subfamily is involved in the development of major depressive disorders. However, the role of BDNF and its functional brain regions involved in PPD remains unknown. Here, we report that chronic unpredictable stress (CUS) can produce depression-associated behaviors in postpartum female mice. CUS can decrease total *Bdnf* mRNA and exon specific mRNAs in the medial prefrontal cortex (mPFC), accompanied by reduced protein levels, that were correlated with depression-related behaviors. Moreover, postpartum, not virgin female mice showed increased susceptibility to subthreshold stress-induced depression-related behaviors. Selective deletion of BDNF in the mPFC induced anhedonia as indicated by reduced sucrose preference and increased latency to food in the novelty suppressed food test in postpartum, but not in virgin female mice. Furthermore, we found that FoxO1 is also decreased in CUS-treated postpartum female mice with a significant correlation with depression-related behaviors. BDNF-specific knockout in the mPFC decreased FoxO1 expression in female mice. Our results indicate that the BDNF-FoxO1 axis in mPFC can regulate depression-related behaviors and stress vulnerability in postpartum female mice.

## Introduction

Postpartum depression (PPD) is a serious mental disorder that can occur in the postpartum period with dramatic physiological and emotional changes in all maternal organisms [1]. PPD is commonly recognized as a subtype of major depressive disorder (MDD), affecting 10–20% of all new mothers within 4 weeks after childbirth [2]. PPD has not only profound adverse effects on mothers but also negatively impacts their infants owing to the absence of maternal care and mother-infant social interactions [3]. In severe situations, patients may even be more likely to commit infanticide and baby abuse [4]. Although the dramatic fluctuation of ovarian hormones during postpartum and the abnormal secretion of glucocorticoids are widely considered as contributors to PPD [5–7], but an association between many other factors involved in energy metabolism, neurodegeneration, and immune response and the pathophysiology of PPD has been identified [8]. Until now, the knowledge about the etiology of PPD is incomplete and the underlying mechanisms are largely unclear.

Brain-derived neurotrophic factor (BDNF) is a secreted neurotrophin that is highly expressed in the central nervous system and regulates many different cellular processes that affect emotional behaviors [9]. The *Bdnf* gene has a complex gene structure containing multiple 5’noncoding exons and a single 3’coding exon to produce multiple exon-specific *Bdnf* transcripts that undergo alternative splicing but encode the same protein [10]. Emerging evidence has indicated the involvement of BDNF in depression, including PPD, on the basis of its roles in pathogenesis and treatment of depression [11–15]. However, the exact function of BDNF in depression of postpartum female mice remains to be investigated.

FoxO1, also named FKHR is a member of the FoxO subfamily, which belongs to the Fox family, a family of transcription factors containing a highly conserved, winged-helix DNA-binding domain and the forkhead motif [16, 17]. FoxO proteins can bind to the regulatory sequence of downstream target genes and play important roles in regulating the transcription of genes involved in multiple biological and pathological systems, including the central nervous system. Recent studies provide evidence for the role of FoxO proteins in the pathogenesis of depression and other psychiatric disorders [18, 19]. FoxO1 is highly expressed in brain areas related to the regulation of mood and stress [20], and FoxO1-deficient mice show a depressive-like phenotype in forced swim test (FST) and tail suspension test (TST) behaviors assessments [18], which means FoxO1 may be involved in the pathology of depression.

Animal models are widely used to study MDD, including PPD. Many laboratory animal models of PPD have been generated through abrupt withdrawal after administering exogenous glucocorticoids or ovarian hormones [21–23], repeated stress during pregnancy [24–26], glucocorticoid exposure, or separating mother from pups during the postpartum period [6, 27], which mimic the contributing biological or psychosocial factors to PPD in women, to induce depressive-like behaviors and altered neuroplasticity or synaptic plasticity in maternal brain areas, such as the prefrontal cortex (PFC), the nucleus accumbens, and the hippocampus [26, 28–31]. However, the response of postpartum female mice to chronic stress-induced depressive behaviors, susceptibility, and the underlying functional genes remain unclear.

Here, we generated an animal model of PPD in which the effects of chronic unpredictable stress on depressive behaviors of postpartum female mice were tested. Additionally, *Bdnf* and *Bdnf*-specific exon mRNA expression in the medial PFC (mPFC) were evaluated and the correlation between depressive behaviors and BDNF expresson levels were analyzed. Next, we measured the susceptibility of postpartum mice to the subthreshold stress. We also analyzed the *FoxO1* mRNA and protein expression and its correlation with depressive behaviors. Finally, we generated mice with conditional BDNF deletion in the mPFC and determined the impact of BDNF loss on depression-related behaviors and FoxO1 expression in mPFC.

## Results

### Chronic unpredictable stress induces depression-related behaviors in postpartum female mice

**Fig. 1:**
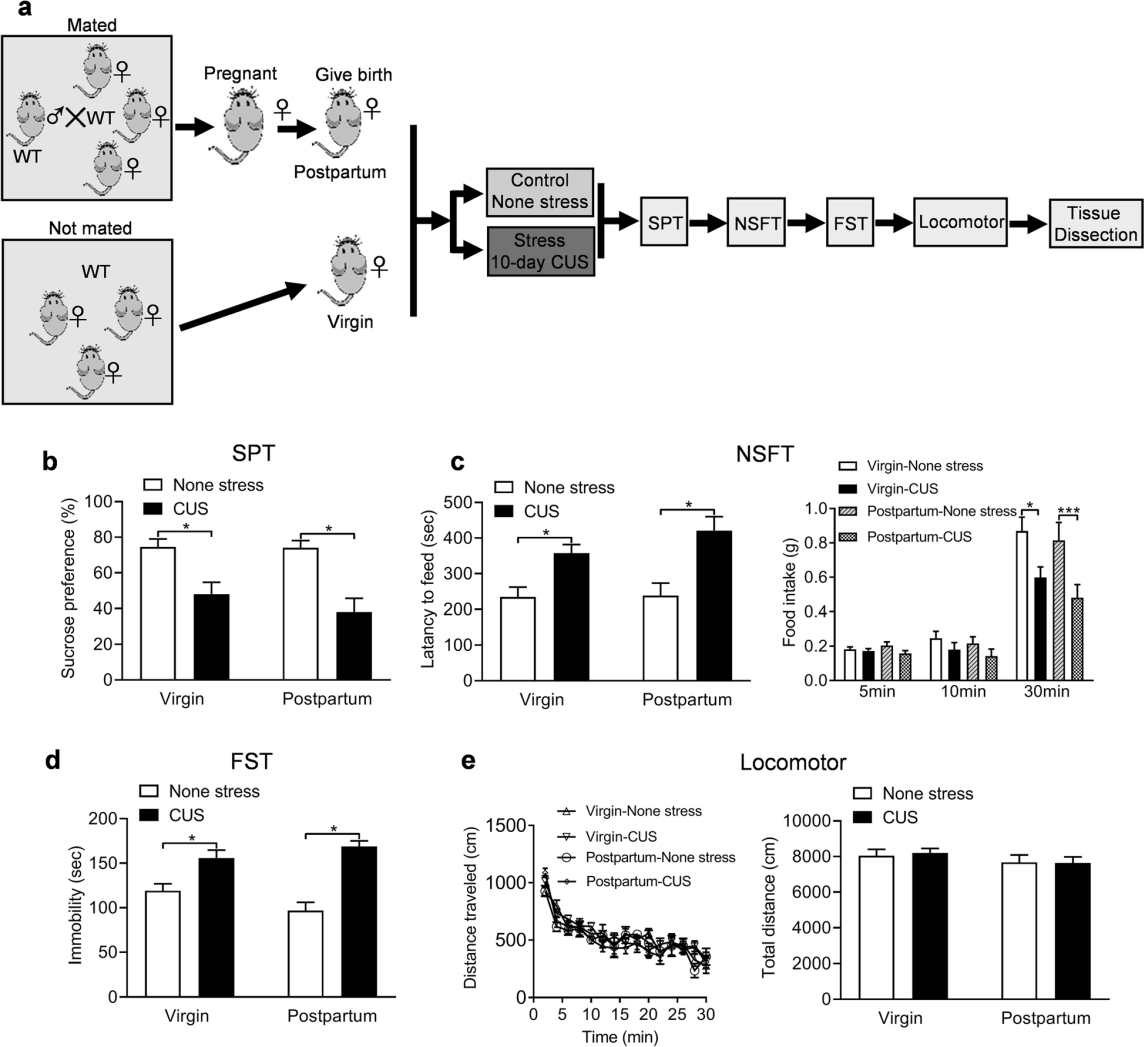
Depression-related behaviors induced by chronic unpredictable stress in virgin and postpartum female mice. **a** Schematic of the experimental timeline. **b** Sucrose preference test (SPT). **c** Novelty-suppressed food test (NSFT). **d** Forced swimming test (FST). **e** Locomotor activity. *p < 0.05, ***p < 0.001 compared with none stress group

CUS is widely used to induce depression in mice [32]. Here, female WT mice were mated with male WT mice. After parturition, the postpartum female mice and virgin female mice were subjected to different stressors for 10 days randomly [33] and the depression-related behaviors were tested after the stresses (Fig. 1a). Anhedonia is a core symptom of depression, which can be assessed by the sucrose preference test (SPT) in mice [34]. We found that CUS dramatically decreased the preference for 1% sucrose when compared with control non-stressed mice in both virgin and postpartum female mice (*P* = 0.024 and *P* = 0.001), and there were no significant difference between virgin and postpartum female mice under none stress and CUS conditions (*P* > 0.999 and *P* = 0.648) (Fig. 1b, fertility condition: *F* (1, 29) = 0.737, *P* = 0.398; stress: *F* (1, 29) = 26.450, *P* < 0.001; fertility condition ×stress interaction: *F* (1, 29) = 0.606, *P* = 0.443). Meanwhile, the novelty-suppressed feeding test (NSFT) was conducted on these mice and the results indicated that the CUS treated virgin and postpartum female mice displayed a significantly increased latency to eat (*P* = 0.042 and *P* = 0.001) (Fig. 1c, fertility condition: *F* (1, 27) = 1.149, *P* = 0.293; stress: *F* (1, 27) = 23.530, *P* < 0.001; fertility condition × stress interaction: *F* (1, 27) = 0.885, *P* = 0.355) and decreased food consumption during the 30-min period (*P* = 0.048 and *P* < 0.001) compared to none stress treated mice, and there were no obvious changes between virgin and postpartum female mice under none stress and CUS conditions (*P* = 0.898 and *P* = 0.388) (Fig. 1c, subgroups: *F* (3, 29) = 3.746, *P* = 0.0217; timepoints: *F* (2, 58) = 184.200, *P* < 0.001; subgroups × timepoints: *F* (6, 58) = 3.865, *P* = 0.003). By the way, increased behavioral despair, as shown by increased immobility in the forced swimming test (FST), was observed in the CUS treated mice as compared with non-stressed mice (*P* = 0.017 and *P* < 0.001) We also did not found significant difference between virgin and postpartum female mice under none stress and CUS conditions (*P* = 0.235 and *P* = 0.672) (Fig. 1d, fertility condition: *F* (1, 29) = 0.359, *P* = 0.554; stress: *F* (1, 29) = 45.390, *P* < 0.001; fertility condition ×stress interaction: *F* (1, 29) = 4.749, *P* = 0.038). To exclude possible effects of non-specific motor activity on mobility, locomotor activity was tested and the results showed no significant difference of distance traveled (Fig. 1e, subgroups: *F* (3, 29) = 0.644, *P* = 0.593; timepoints: *F* (14, 406) = 35.790, *P* < 0.001; subgroups × timepoints: *F* (42, 406) = 1.016, *P* = 0.447) and total distance (*P* = 0.998 and *P* = 0.999) (subgroups: *F* (1, 29) = 0.034, *P* = 0.855; timepoints: *F* (1, 29) = 1.812, *P* = 0.189; subgroups × timepoints: *F* (1, 29) = 0.078, *P* = 0.782) in CUS treated virgin and postpartum female mice comparing with non-stressed mice.

### Expression of *Bdnf* in the mPFC is down-regulated in postpartum female mice

**Fig. 2:**
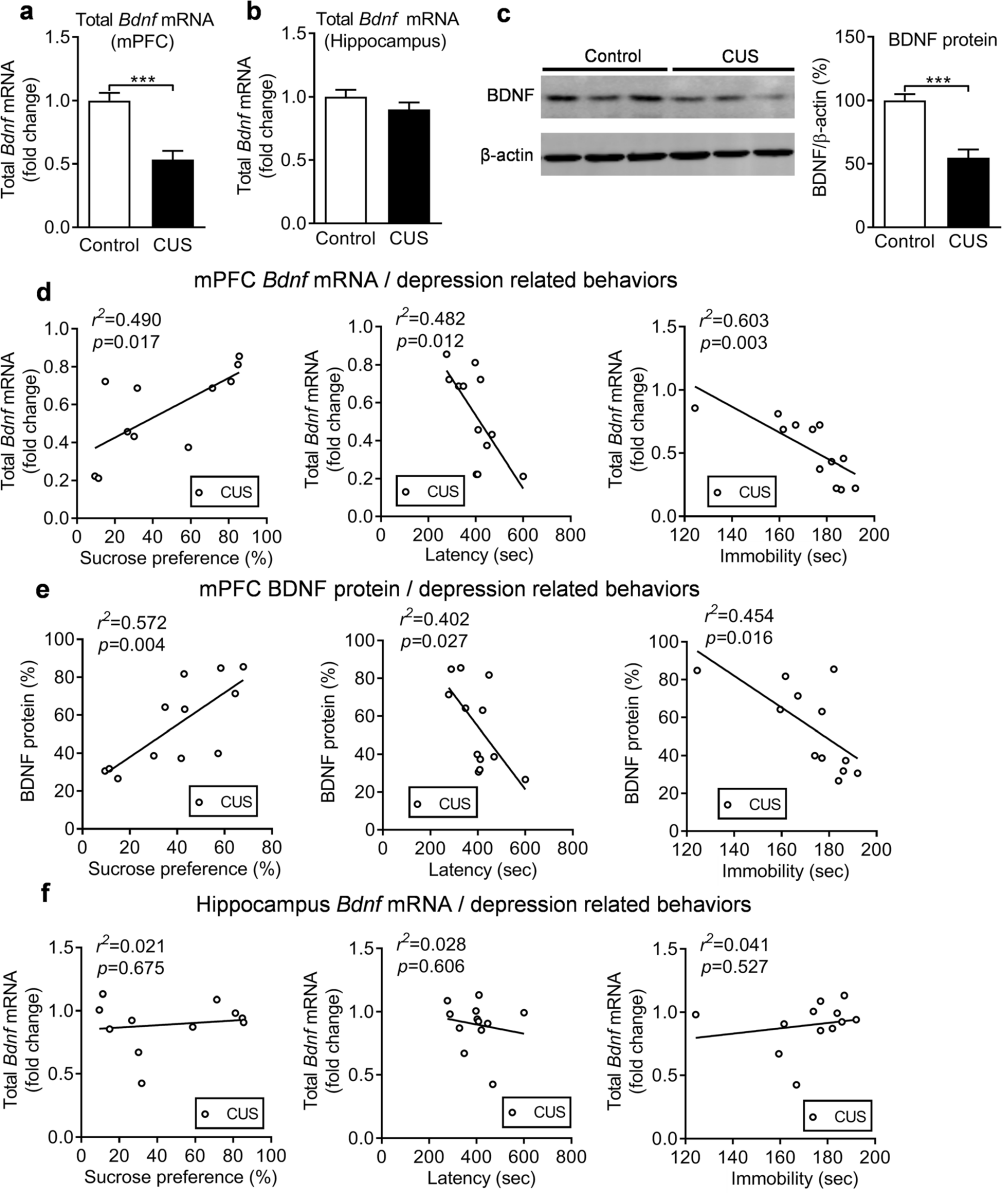
Regulation of BDNF expression by chronic unpredictable stress. Total *Bdnf* mRNA levels in the mPFC (**a**) and the hippocampus (**b**) of control and CUS groups. **c** Immunoblots showing BDNF protein levels in the mPFC of control and CUS groups. **d** Correlation analysis between SPT, NSFT, or FST and total *Bdnf* mRNA levels in the mPFC. **e** Correlation analysis between, SPT, NSFT, or FST and BDNF protein levels in mPFC of CUS mice. **f** Correlation analysis between SPT, NSFT, or FST and total *Bdnf* mRNA levels in the hippocampus of CUS mice. ***p < 0.001 compared with control group

The mPFC and hippocampus have an important role in the pathogenesis of MDD [35, 36]. To assess whether BDNF is involved in PPD in female mice, we first measured total *Bdnf* mRNA levels in CUS-treated postpartum female mice. The results indicated that total *Bdnf* mRNA (exon IX) levels were significantly decreased in the mPFC (Fig. 2a*, P* < 0.001) but not in the hippocampus (Fig. 2b*, P* = 0.215). Meanwhile, BDNF protein levels in the mPFC were also significantly reduced in CUS-treated postpartum mice compared to non-stressed mice (Fig. 2c*, P* < 0.001). Furthermore, the correlation between depression-related behavior and BDNF expression levels was analyzed and we found that sucrose preference (SPT) (*p* = 0.017), latency (NSFT) (*p* = 0.012), and immobility (FST) (*p* = 0.003) were significantly correlated with total *Bdnf* mRNA in the mPFC (Fig. 2d). At the same time, significant correlations were also observed between sucrose preference (*p* = 0.004), latency (*p* = 0.027), and immobility (*p* = 0.016) and BDNF protein levels in the mPFC (Fig. 2e). However, there were no significant correlations between sucrose preference (*p* = 0.675), latency (*p* = 0.606), and immobility (*p* = 0.527) and *Bdnf* mRNA levels in the hippocampus (Fig. 2f). The *Bdnf* gene contains multiple exon-specific transcripts. Therefore, we measured the *Bdnf*-specific exon mRNA levels in the mPFC of CUS-treated postpartum mice. The results show that exon I, exon II and exon VI levels were significantly decreased, while exon III and exon IV levels were unchanged (Additional file 1: Supplementary Fig. 1, Exon I: *p* = 0.028; Exon II: *p* = 0.028; Exon III: *p* = 0.165; Exon IV: *p* = 0.227; Exon VI: *p* = 0.003).

### Postpartum female mice show increased susceptibility to SCUS-induced depression-related behaviors

**Fig. 3:**
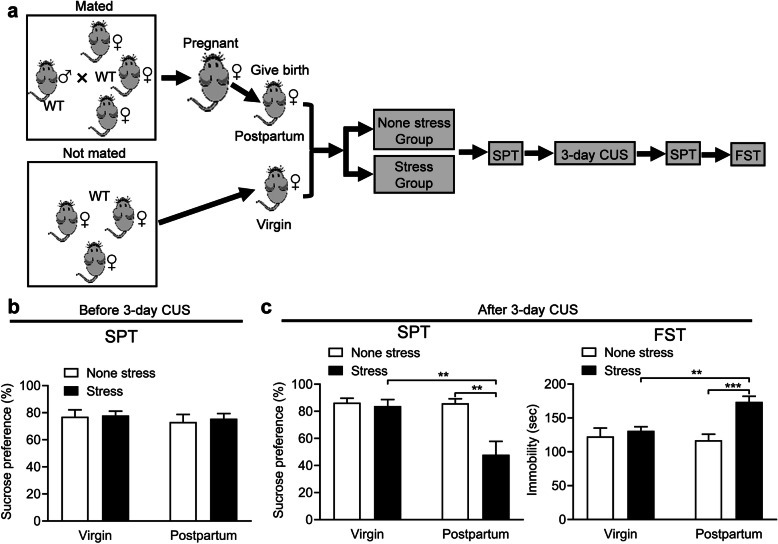
Susceptibility to subthreshold stress-induced depression-related behavior in postpartum female mice. **a** Schematic of the experimental timeline. **b** Sucrose preference test before stress exposure of virgin and postpartum female mice. **c** Sucrose preference test (SPT) and forced swimming test (FST) after stress exposure of virgin and postpartum female mice. **p < 0.01, ***p < 0.001 compared with controls

Stress susceptibility is an important aspect of depression. Thus, we evaluated the susceptibility of postpartum female mice to Subchronic unpredictable stress (SCUS). Both postpartum female mice and virgin female mice were divided into two groups: non-stressed and stressed groups. Mice in the stressed group were subjected to 3 days of CUS (Fig. 3a). SPT was conducted before and after stress exposure. Before stress exposure, a two-way ANOVA revealed no effect of fertility condition (*F* (1, 30) = 0.477, *P* = 0.495), stress (*F* (1, 30) = 0.132, *P* = 0.719) and fertility condition × stress interaction (*F* (1, 30) = 0.031, *P* = 0.037) on sucrose preference, showing that the basal depressive-like behaviors of virgin and postpartum female mice were similar (Fig. 3b). After stress exposure, there were significant effects of the fertility condition (*F* (1, 30) = 7.311, *P* = 0.011), stress (*F* (1, 30) = 9.080, *P* = 0.005) and fertility condition × stress interaction (*F* (1, 30) = 6.980, *P* = 0.013) on sucrose preference. Virgin mice showed no changes of preference for sucrose (*P* = 0.995), while postpartum mice demonstrated reduced (*P* = 0.001) preference in the SPT compared to non-stressed mice. Meanwhile, the preference for sucrose was also reduced in stressed postpartum mice compared to stressed virgin mice (*P* = 0.003) (Fig. 3c). We also found a significant effect of fertility condition (*F* (1, 30) = 4.288, *P* = 0.047), stress (*F* (1, 30) = 13.350, *P* = 0.011) and fertility condition × stress interaction (*F* (1, 30) = 7.404, *P* = 0.013) on immobility in the FST. Further analysis indicated that stressed virgin mice had no significant immobility time (*P* = 0.924), while stressed postpartum mice had more immobility time (*P* < 0.001) compared to non-stressed mice. The immobility time was also elevated in stressed postpartum mice compared to stressed virgin mice (*P* = 0.008) (Fig. 3c).

### Deletion of *Bdnf* in the mPFC produces depression-related behaviors in postpartum female mice

**Fig. 4:**
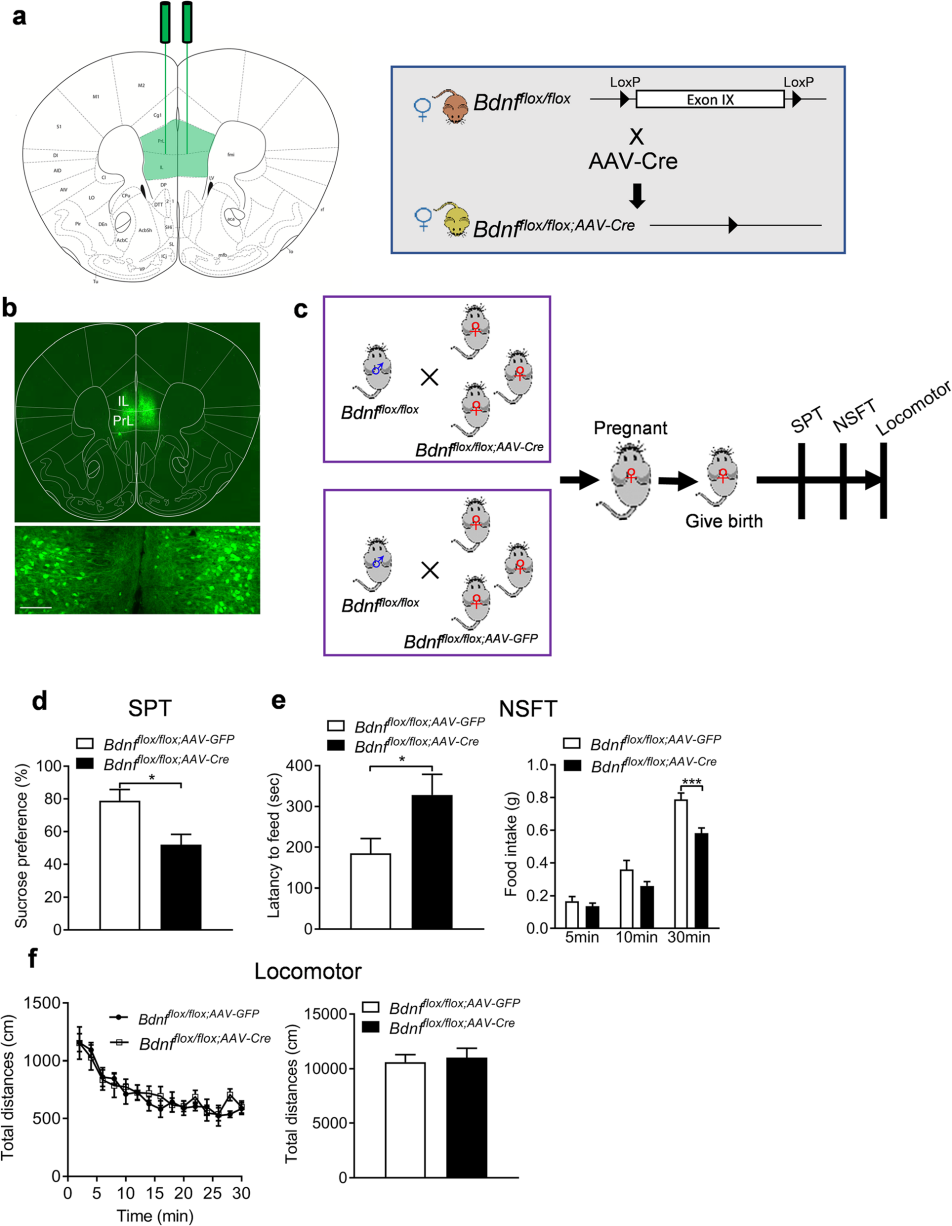
Selective deletion of *Bdnf* in the mPFC induces depression-related behaviors in postpartum female mice. **a** Schematic illustration of stereotaxic injection of AAV-Cre or AAV-GFP vectors into the mPFC. **b** Representative image showing GFP expression at the injection sites. Sale bars = 100 μm. **c** Schematic of the experimental timeline. **d** Sucrose preference test. **e** Novelty-suppressed food test. **f** Locomotor activity. *p < 0.05, ***p < 0.001 compared with AAV-GFP group

We found that *Bdnf* expression in the mPFC was decreased in CUS postpartum female mice, and the critical role of the mPFC in the pathophysiology of depression is well known [35]. Consequently, we hypothesized that BDNF in the mPFC modulates depression-like behaviors. We used Cre recombinase-expressing AAV vectors to selectively delete the loxP-flanked *Bdnf* gene. The region-specific BDNF knockdown in the mPFC was achieved by bilateral intra-mPFC injection of AAV-Cre into adult female *Bdnf*^*flox/flox*^ mice (Fig. 4a). Three weeks later, we confirmed the injection site and transfection of AAV in the mPFC (Fig. 4b). We first evaluated the basal depression behaviors of these mPFC-specific BDNF knockout (*Bdnf*^*flox/flox;AAV-Cre*^) and control mice (*Bdnf*^*flox/flox;AAV-GFP*^) under none stress conditions (Additional file 2: Supplementary Fig. 2a), and the results showed that mice injected with AAV-Cre showed no difference in preference for the 1% sucrose solution compared with AAV-GFP injected control mice (Additional file 2: Supplementary Fig. 2b, *P* = 0.784). The immobility of the AAV-Cre injected mice in the FST is comparable to the AAV-GFP injected mice (Additional file 2: Supplementary Fig. 2c, *P* = 0.830). Meanwhile, there were no changes of the latency to eat (*P* = 0.565) and food consumption (5 min: *P* = 0.969, 10 min: *P* = 0.981, 30 min: *P* = 0.423) of AAV-Cre injected mice in the NSFT compared to mice injected with the control virus (Additional file 2: Supplementary Fig. 2d*,* genotype: *F* (1, 18) = 0.649, *P* = 0.431; timepoints: *F* (2, 36) = 90.480, *P* < 0.001; genotype × timepoints: *F* (2, 36) = 1.116, *P* = 0.339). Then, the virus-injected female mice were mated with male *Bdnf*^*flox/flox*^ mice. After parturition, the postpartum female mice were used for behavioral assessments (Fig. 4c). In the SPT, mice injected with AAV-Cre showed a decrease in preference for the 1% sucrose solution compared with AAV-GFP injected control mice (Fig. 4d, *P* = 0.012). Additionally, the latency to eat (*P* = 0.024) was increased, while food consumption during the 30-min period (*P* = 0.001) was significantly increased of AAV-Cre injected mice in the NSFT compared to mice injected with the AAV-GFP virus (Fig. 4e*,* genotype: *F* (1, 14) = 9.122, *P* = 0.009; timepoints: *F* (2, 28) = 235.300, *P* < 0.0001; genotype × timepoints: *F* (2, 28) = 6.132, *P* = 0.006). Finally, there were no differences in locomotor activity of AAV-Cre and AAV-GFP injected mice (Fig. 4f, distance travelled: genotype: *F* (1, 14) = 0.073, *P* = 0.792; timepoints: *F* (14, 196) = 23.500, *P* < 0.001; genotype × timepoints: *F* (14, 196) = 1.272, *P* = 0.851; total distance: *P* = 0.734).

### CUS decreases *FoxO1* expression in the mPFC in postpartum female mice

**Fig. 5:**
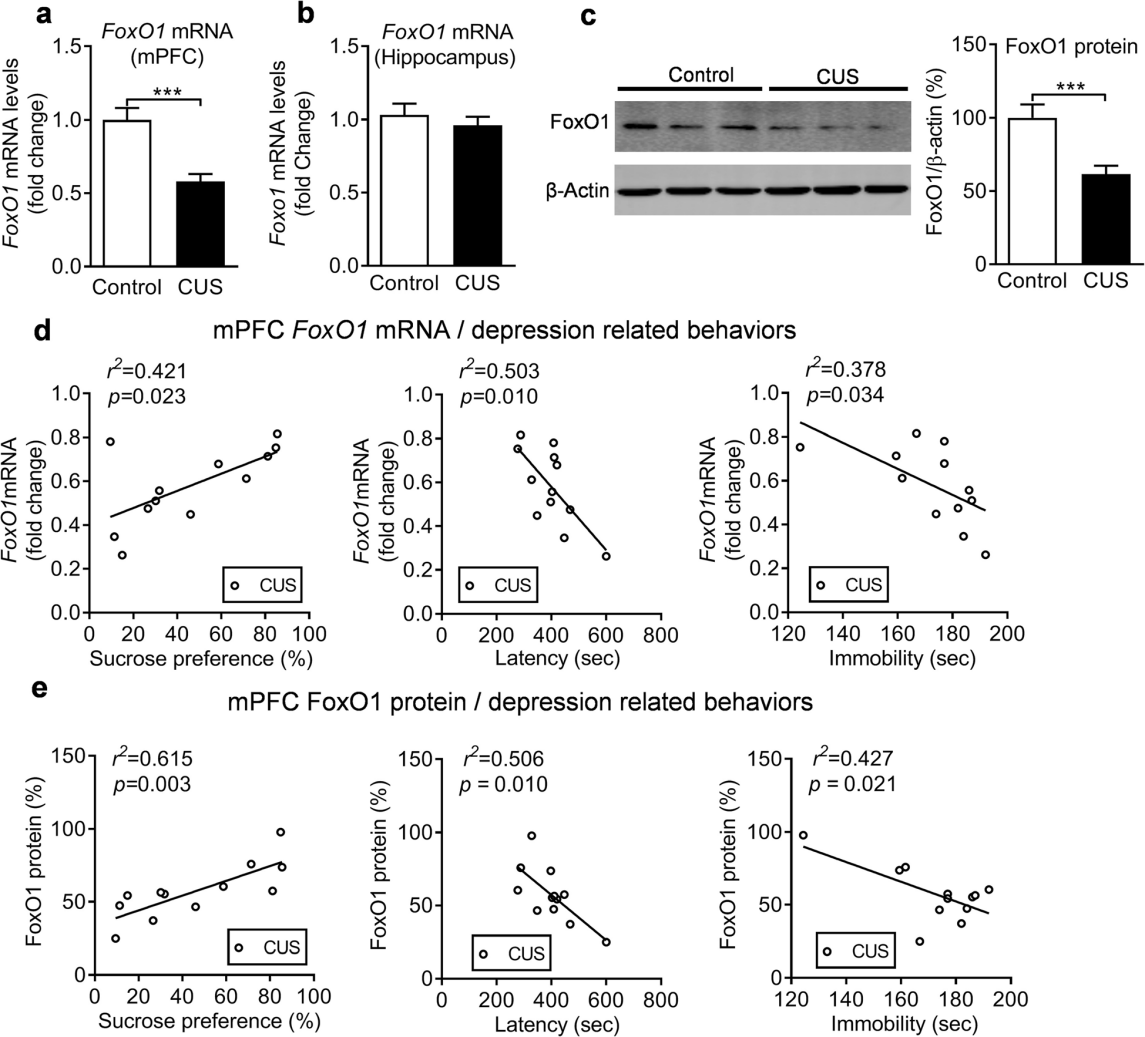
Regulation of FoxO1 expression by chronic unpredictable stress. FoxO1 mRNA levels in the mPFC (**a**) and the hippocampus (**b**) of control and CUS groups. **c** Immunoblots showing FoxO1 protein levels in the mPFC of control and CUS groups. **d** Correlation analysis between SPT, NSFT, or FST and *FoxO1* mRNA levels in the mPFC of CUS mice. **e** Correlation analysis between, SPT, NSFT, or FST and FoxO1 protein levels in the mPFC of CUS mice. ***p < 0.001 compared with control group

To investigate the underlying role of BDNF on postpartum depression in female mice, and given a study reporting that FoxO1 is a underlying functional target gene of *Bdnf* [37]. We measured *FoxO1* mRNA levels in CUS-treated female mice and found that *FoxO1* mRNA levels were significantly reduced in the mPFC (Fig. 5a*, P* < 0.001) but not in the hippocampus (Fig. 5b*, P* = 0.461). Moreover, FoxO1 protein levels in the mPFC were also decreased in CUS-treated postpartum mice compared to non-treated mice (Fig. 5c*, P* = 0.001). The correlation analysis revealed that there were significant positive or negative correlations between depression-related behavior and *FoxO1* mRNA expression levels (Fig. 5d). At the same time, significant correlations were also observed between sucrose preference, latency or immobility and FoxO1 protein levels in the mPFC (Fig. 5e).

### FoxO1 expression in the mPFC can be regulated by BDNF

**Fig. 6:**
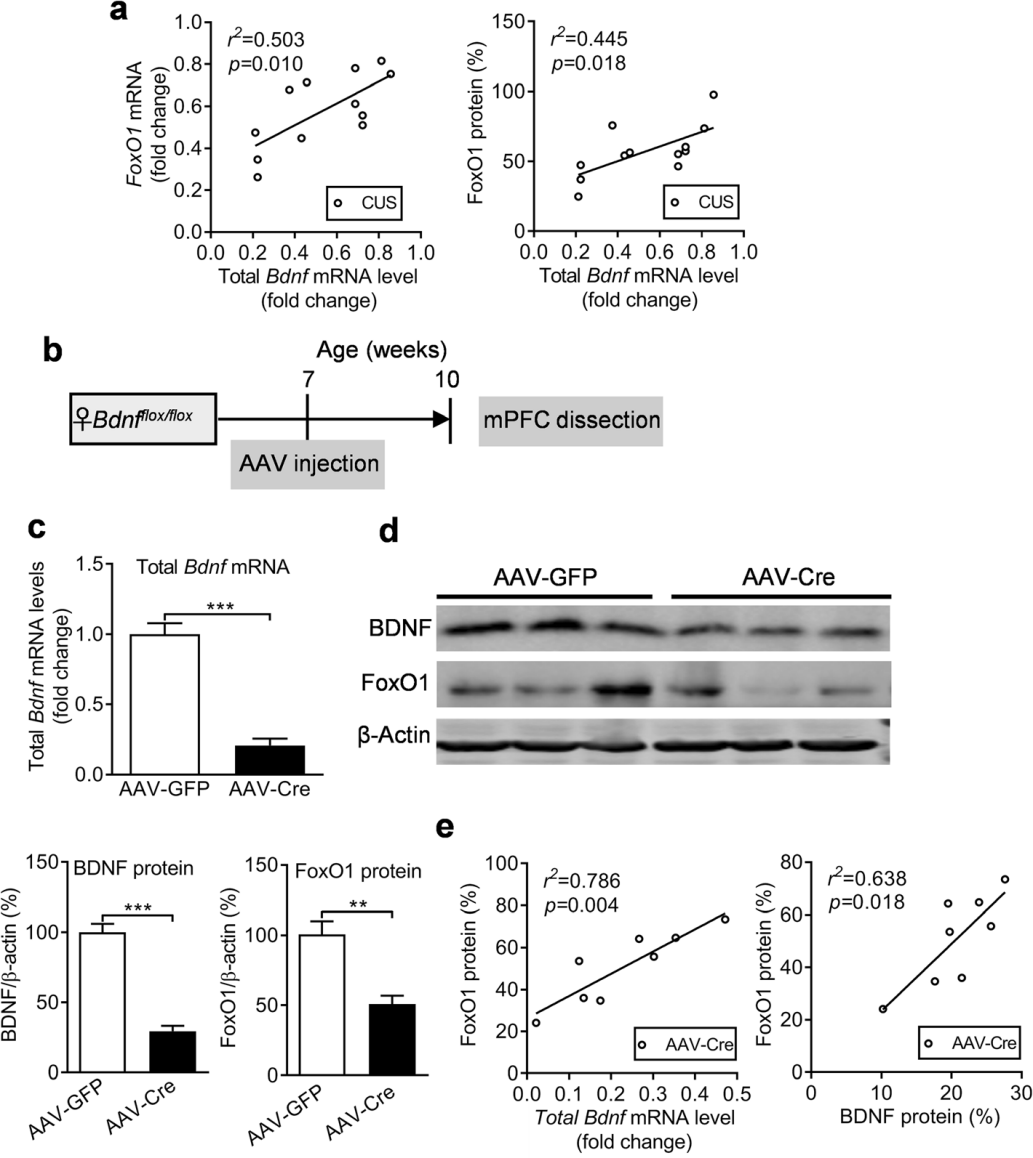
FoxO1 expression level is regulated by BDNF in the mPFC. **a** Correlation analysis between total *Bdnf* mRNA level and FoxO1 mRNA or protein levels in the mPFC of CUS treated postpartum female mice. **b** Schematic of the experimental timeline. **c** Total *Bdnf* mRNA level after *Bdnf* deletion in the mPFC. **d** Immunoblots showing BDNF and FoxO1 protein levels in the mPFC of the AAV-GFP and AAV-Cre groups. **e** Correlation analysis between total *Bdnf* mRNA or protein levels and FoxO1 protein levels. **p < 0.01, ***p < 0.001 compared with AAV-GFP group

To study the regulatory relation of BDNF and FoxO1 in postpartum female mice, we conducted a correlation analysis between BDNF and FoxO1 levels in mPFC of CUS treated postpartum female mice, which indicated that total *Bdnf* mRNA levels were significantly positive correlated with FoxO1 in both mRNA (*P* = 0.010) and protein (*P* = 0.018) levels (Fig. 6a). Moreover, to determine whether FoxO1 can be regulated by BDNF in the mPFC, we injected AAV-Cre or AAV-GFP vectors bilaterally into the mPFC to delete *Bdnf* in the mPFC. Three weeks later, mPFC tissue was dissected for further analysis (Fig. 6b). Quantitative real-time PCR results demonstrated that total *Bdnf mRNA* levels were significantly reduced by AAV-mediated knockdown (Fig. 6c, *P* < 0.001). Furthermore, BDNF and FoxO1 protein levels were also significantly decreased in AAV-Cre-GFP-injected mice compared to AAV-GFP-injected control mice (Fig. 6d, BDNF: *P* < 0.001; FoxO1: *P* = 0.009). We also found a positive correlation between total *Bdnf* mRNA (*P* = 0.004) or protein (*P* = 0.018) levels with FoxO1 protein level in the AAV-Cre treated mice respectively (Fig. 6e).

## Discussion

In this study, we showed that the BDNF-FoxO1 axis in the mPFC is important for regulating depression-related behavior in postpartum female mice. We found that CUS can induce depression-related behavior in postpartum female mice and that a *Bdnf*-specific exon mRNA expression was decreased in the mPFC of female mice with depression-like behavior, resulting in low expression of total *Bdnf* mRNA that coincided with depression-related behaviors. The important finding was that postpartum female mice showed higher susceptibility to SCUS-induced depression-related behavior and that mPFC-specific BDNF-knockout induced differential behavioral phenotypes in virgin and postpartum female mice. Reduced FoxO1 levels in the mPFC were also involved in depression-related behaviors of CUS-treated postpartum female mice. Furthermore, mPFC-specific BDNF knockout also resulted in decreased modulation of FoxO1 in the mPFC. Collectively, these data provide strong evidence for the neuronal role of BDNF in PPD.

Several animal models have been established to study the development of and treatment for PPD. The most widely used PPD models are the ovarian-steroid-withdrawal model [22], corticosterone-induced model [23], and gestational stress (restraint stress) [24]. Disadvantages of these models include their inability to assess the effects of parturition and the response to multiple stress exposures after parturition which is considered as the leading factor in 85% of cases of depression in patients [38]. Here, we showed female mice subjected to a 10 days CUS after parturition can mimic puerpera suffering from stress. Our established depression model induced depression-like behavioral deficits in postpartum and virgin female mice i.e. anhedonia and behavioral despair as indicated by decreased sucrose preference, increased latency to food, and immobility in FST, which is consistent with previous findings in male mice [33, 39], however, there are no obvious difference between postpartum and virgin female mice, which means the unchanged susceptibility under the 10 days CUS paradigm.

The incidence of depression is 2–3-fold higher in women than in men [40]. In particular the postpartum period is considered the time of greatest risk for women to develop MDD [41]. In order to address response of susceptibility in postpartum female mice, we applied a short term SCUS (3 days CUS), which is the usually used strategy to evaluate the susceptibility to stress [42, 43]. Our findings demonstrated that female mice after parturition showed similar depression-like phenotypes to virgin females, which means the parturition stress alone cannot induce depression, while postpartum female mice showed enhanced susceptibility to SCUS induced depression-related behavior compared to virgin females, which reminds that 3 days stress may be the threshold of time window for stress induced depressive behaviors in PPD model. The reason for the higher susceptibility to stress of postpartum female mice may be due to the hormonal fluctuations during pregnancy and postpartum [4]. One indication for this is the hypothesis of ovarian-steroid-withdrawal as the cause of postpartum depression [22, 44]. Estrogen levels increase by over 1000-fold shortly before parturition compared to their normal values and then drop dramatically after parturition [45]. This sudden drop in estradiol and progesterone is believed to be an important factor in the postpartum blues, a phenomenon which is observed in 80% of women shortly after birth and which can convert to PPD [5, 46]. This is consistent with a report that women suffering from polycystic ovary syndrome, which is associated with low estrogen levels, have an increased risk for mood disorders [47]. These findings suggest that low levels or sudden declines in estradiol and/or progesterone levels may predispose vulnerable women to depression. Besides the fluctuations in gonadal hormones during pregnancy and postpartum, it is important to note that the hypothalamic–pituitary–adrenal axis (HPA) also undergoes significant changes in the postpartum period. For example, pregnancy-related steroid hormones and peptides, such as oxytocin, dampen the reactivity of the HPA axis during pregnancy. Furthermore, there is evidence that cortisol, adreno-cortico-tropic-hormone, corticotropin releasing hormone, and corticosterone binding globulin (CBG) levels are altered significantly during pregnancy and postpartum. For example, stress hormone levels rise during pregnancy, reach a peak before birth, and drop after birth in rats and humans [45]. Based on this fact, corticosterone has been used to establish a PPD animal model [27]. In summary, the postpartum period is a time of increased vulnerability to depression which may be due to the dramatic fluctuations of gonadal and adrenal hormones that deserve special attention and consideration. However, more research is needed to better understand the causes and contributing factors for depression during postpartum.

Many experiments suggest that BDNF is a key gene in depression, Notably, lower BDNF levels are found in the hippocampus and the PFC of subjects who committed suicide compared with non-suicide controls, whereas higher BDNF levels are observed in individuals treated with antidepressants compared with drug-free controls [48, 49]. Our results showed that mRNA and protein levels of BDNF were decreased in the mPFC but not in the hippocampus of CUS-treated postpartum mice, which indicates a brain-region specific function. Furthermore, Pearson’s test analysis showed that total *Bdnf* mRNA in the mPFC was significantly correlated to the depression-related behavior. These findings suggest that suppression of BDNF expression in the mPFC may be involved in the pathology of PPD, which is in accordance with the viewpoint that mPFC is one of the crucial regions for the pathology of depression. However, mPFC specific BDNF deletion can induce depression-related behaviors in postpartum only, but not in virgin female mice, which suggests the specific role of *Bdnf* in mPFC on PPD. A recent study showed that T cell death-associated gene 51 (TDAG51) knockout, microglia-specific autophagy-deficient mice combined with chronic unpredictable mild stress during gestation also can induce depression-related behaviors in the female mice after parturition also support the fact that certain functional genes indeed can affect the behaviors in the postpartum female mice [12, 50]. The *Bdnf* gene contains multiple promotor-specific exon mRNAs [10] to encode exactly the same protein. Our results indicated that exon I, II, and VI were down-regulated by CUS, which is in contrast to previous reports that chronic social defeat stress reduces exon IV and VI expression in the hippocampus of male mice [51]. This suggests a different and complex modulation of BDNF-specific promoters in female and male subjects with depression, however, the particular modifications of these promoters activity needs further investigation.

FoxOs subfamily proteins are involved in the development of MDD [19]. Our findings also indicate that PPD female mice also showed decreased FoxO1 expression in the mPFC but not in the hippocampus and a significant correlation of FoxO1 expression with depression-related behaviors. Additionally, a study using a FoxO1 knockout mouse model suggested that FoxO1 KO mice displayed depression-like behaviors as indicated by behavioral despair in the FST and TST [18]. All of these facts indicate that the inhibitory expression of FoxO1 is associated with depression. The correlation between BDNF and FoxO1 in PPD female mice shows that FoxO1 may be an underlying functional target gene of BDNF. The transcriptional activity of FoxO genes usually can be regulated by BDNF through activation of TrkB and downstream kinases [37, 52]. However, BDNF showed no effect on the phosphorylation of FoxO1 protein and its translocation in PC12 cells [53], which indicates that the regulatory mechanism of BDNF on the activity of FoxO1 may not be associated with protein modification level. Our results also demonstrated that specific BDNF knockout can reduce FoxO1 expression, showing that BDNF can affect FoxO1 activity by gene transcription/translation regulation. However, the underlying mechanism seems to be complicated, as BDNF can activate multiple signaling pathways (PI3K/AKT, MEK/ERK, PLCγ-PKC and PLCγ-CaMKII, etc.) to regulate certain transcription factors (CREB and NF-κB, etc.) by inducing TrkB receptor phosphorylation [54], but the exact functional mechanism needs to be revealed in the future.

In conclusion, our results demonstrate that BDNF in the mPFC is involved in CUS-induced depression-related behavior and increased stress susceptibility in postpartum female mice and postpartum-specific BDNF deletion revealed an important regulatory role of BDNF in depression-related behavior. FoxO1 may participate in the functional regulation of BDNF in this process. Our observations provide a basis for a novel neurobiological mechanism for the pathology and treatment of PPD.

## Methods

### Animals

Wild-type (WT) C57BL/6 J male and female mice (Stock No. 000664) and *Bdnf*^*flox/flox*^ (Stock No. 004339) mice were purchased from Jackson Laboratory (Bar Harbor, ME, USA). *Bdnf*^*flox/flox*^ mice [55], which were generated by loxP sites flanking exon 9 of the *Bdnf* gene, were backcrossed to C57BL/6 J for at least 8–10 generations to generate a common genetic background. The PCR primers used for genotyping were as follows: *Bdnf* WT and flox, forward-5′-TGTGATTGTGTTTCTGGTGAC-3′, reverse-5′-GCCTTCATGCAACCGAAGTATG-3. All mice were housed in groups of five per cage under a 12-h light/dark cycle (lights on at 7:00 am) with ad libitum access to water and standard food pellets, except when explicitly stated differently for behavioral testing. For the experiments, both male and female mice aged between 7 and 15 weeks were used. This study was approved by the Institutional Animal Care and Use Committee of the Binzhou Medical University Hospital and was conducted in accordance with the National Institute of Health Guide for the Care and Use of Laboratory Animals (NIH Publications No. 80–23) revised 1996.

### Postpartum depression model

Three female mice were housed with one male of the same genotype in one cage. The average time for impregnation was 1 week. After that, the male was removed from the cage. About 3 weeks later, the mated females gave birth, their pups were removed, and the postpartum female mice in each cage were divided into two groups separately, one group (stress) was subjected to 10 days of chronic unpredictable stress (CUS), which was performed as described previously [33] and consisted of eight different stressors over 10 days. There were two different stressors daily at different times of the day to prevent stress habituation. Stressors were administered as follows: two-hour restraint, 15-min tail pinch, 24-h constant light, 24-h wet bedding, 45° cage tilt, ten-minute inescapable foot shocks, 30-min elevated platform, and social isolation. The other group (control) was handled daily. In some experiments, an abbreviated (3 days) subthreshold chronic unpredictable stress was used to assess stress susceptibility in normal control and postpartum female mice [43].

### Behavioral assessments

All behavioral measurements were conducted in the late light cycle except the sucrose preference tests which were conducted in the first 2 h of the dark cycle. The behavior of each mouse was scored by persons who were blind to the treatment.

### Sucrose preference test

Testing was conducted according to previously published protocols [43]. Mice were habituated to drinking water from two stoppers fitted with ball-point sipper 50-ml tubes for at least 1 week before testing. Mice were individually housed in the behavioral testing room 4 h before testing and then provided with a free choice between plain water and 1% sucrose during the first 2 h after lights were switched off in the dark cycle. The amount of water and sucrose consumed was evaluated by weight. The preference for sucrose solution was calculated by determining the percentage of total sucrose consumption volume divided by total liquid consumption volume (sucrose + water). For mice subjected to 3 days of subchronic unpredictable stress, the sucrose preference test was performed 24 h after the last stressor.

### Novelty suppressed food test

This test was adapted from a published protocol [56]. Mice were food-deprived 24 h before testing. On the day of testing, mice were habituated to the testing room for 2–3 h and were then placed into a plastic box (50 × 50 × 20 cm^3^) with bedding in a quiet room with dim lighting. A single pellet of food was placed in the center of the box. Then the mice were placed in the corner of the box and the latency to eat was scored for 10 min. After testing, the mice were put back to their home cage and the food intake was calculated at 5 min, 10 min, and 30 min.

### Forced swimming test

The forced swimming test was conducted as described previously [43, 57]. Briefly, mice were tested in a clear Plexiglas cylinder (25 cm high; 10 cm in diameter) containing 15-cm deep, 24 °C warm water. The behavior was videotaped by a charge-coupled device camera positioned directly above the cylinder. The immobility time which was defined as no movement except those caused by respiration of each mouse in last 4 minof the total six period was scored.

### Locomotor activity

The locomotor activity was tested as described previously [33, 43, 57] and measured in the SuperFlex Fusion open field cages (40 × 40 × 30 cm, Omnitech Electronics Inc., Columbus, OH). The mice were acclimated to the testing room for 2–3 h and were then allowed to freely explore for 30 min under the illuminated conditions. The movements of mice were monitored by infrared photosensors on the cage and the total distance traveled was analyzed by the Fusion software (Omnitech Electronics Inc., Columbus, OH).

### Stereotaxic surgery

Intra-mPFC virus injection was performed under anesthesia as previously described [33]. Briefly, AAV9-CMV-Cre-GFP (referred to as AAV-Cre) containing the genes for Cre recombinase and green fluorescent protein (GFP), and control AAV9-CMV-GFP (AAV-GFP) containing the GFP gene alone with titers > 1 × 10^12^ vg/mL (UNC Vector Core, Chapel Hill, NC) were injected bilaterally into the mPFC (coordinates: AP = + 1.8 mm, ML = ± 0.4 mm, DV = − 2.6 mm from the bregma) of adult female *Bdnf*^*flox/flox*^ mice (7 weeks old). A total volume of 0.5 μL adeno-associated virus (AAV) vectors (per side) was delivered at a rate of 0.10 μL/minute with a 33-gauge stainless steel injector connected to a UMP3 microsyringe pump (World Precision Instruments, Sarasota, FL). Additional 10 min were allowed for diffusion and prevention of backflow. Behavioral tests were performed 21 days after AAV injection. The injection sites and the Cre efficiency were verified in each animal at the end of the experiment. Mice with “untargeted” injections were excluded from statistical analysis.

### Quantitative real-time PCR analysis

Mice were decapitated and the brains were removed rapidly. The mPFC and hippocampus were dissected on ice. All protocols are described elsewhere [33, 43, 57]. Briefly, total RNA was extracted using a tissue RNA kit (Omega, Guangzhou, China) following the manufacturer’s instructions. Total RNA was reversely transcribed into cDNA using HiScript II Q RT SuperMix for qPCR (+ gDNA wiper) kit (Vazyme, Nanjing, China) with gDNA wiper to remove genomic DNA contamination according to the manufacturer’s instructions. The resulting cDNA was used for real-time PCR detection using the StepOnePlus real-time PCR system (Applied Biosystems, Waltham, MA, USA). The primer sequences used to amplify each gene were as follows [58, 59]: *Bdnf* exon IX forward: GCGCCCATGAAAGAAGTAAA; reverse: TCGTCAGACCTCTCGAACCT, *Bdnf* exon I forward: CCTGCATCTGTTGGGGAGAC; reverse: GCCTTGTCCGTGGACGTTTA, *Bdnf* exon II forward: CTAGCCACCGGGGTGGTGTAA; reverse: AGGATGGTCATCACTCTTCTC, *Bdnf* exon III forward: CTTCATTGAGCCCAGGTCC; reverse: CCGTGGACGTTTACTTCTTTC, *Bdnf* exon IV forward: CAGAGCAGCTGCCTTGATGTT; reverse: GCCTTGTCCGTGGACGTTTA, *Bdnf* exon VI forward: CTGGGAGGCTTTGATGAGAC; reverse: GCCTTCATGCAA CCGAAGTA, FoxO1 forward: TTCAATTCGCCACAATCTGTCC; reverse: GGGTGATTTTCCGCTCTTGC. *β-tubulin* forward: AGCAACATGAATGACCTGGTG; reverse: GCTTTCCCTAACCTGCTTGG, the housekeeping gene *β-tubulin* was used as a reference gene for normalization of gene expression. The 2^-ΔΔCT^ method, i.e. delta-delta-ct analysis, was used for relative quantification [43, 57, 60].

### Western blot assay

Western blots were performed as described previously [43]. Briefly, the dissected mPFC samples were homogenized in a lysis buffer (Beyotime Biotechnology, Shanghai, China) with 1% phenylmethylsulfonyl fluoride (Sangon Biotech, Shanghai, China) and 1 × PhosSTOP phosphatase inhibitor cocktail (Roche Applied Science, Penzberg, Germany). The extracted proteins were separated on a 15% SDS-PAGE gel and transferred to PVDF membrane (Millipore, Massachusetts, USA). The membrane was blocked with 5% non-fat milk powder in TBST buffer (20 mM Tris-HCl, pH 7.4, 150 mM NaCl, 0.1% Tween20), followed by incubation with the following primary antibodies diluted in a blocking solution overnight at 4 °C: anti-BDNF (sc-546, 1:500, Santa Cruz Biotechnology, Dallas, TX, USA), anti-FoxO1 (1:500, #2880, Cell Signaling Technology, Danvers, MA, USA), or anti-β-actin antibody (1:1000, #4970, Cell Signaling Technology, Danvers, MA, USA). After washing, the membrane was incubated with IRDye 680LT donkey anti-rabbit IgG secondary antibodies (1:5000; 926–68,023, Li-COR Biosciences, Lincoln, NE, USA). The fluorescence was visualized and analyzed using an Odyssey Infrared Imaging System (Li-COR Biosciences, Lincoln, NE, USA).

### Statistical analyses

All statistical analyses were performed using the statistical software GraphPad Prism 7. All data are presented as mean ± standard error (s.e.m.). Shapiro-Wilk tests and F tests were used to test the normality and equal variance assumptions, respectively. Two-tailed t-tests were used to assess differences between two experimental groups with equal variance. For multiple groups, two-way or two-way repeated-measures ANOVAs followed by Turkey tests were used. Statistical significance was set as *p* < 0.05. Grubbs outlier test was performed and samples that varied > 2 standard deviations from the mean were removed.

## Supplementary information

**Additional file 1 : Supplementary Fig. 1.** Regulation of *Bdnf* exon mRNA expression in the mPFC by chronic unpredictable stress. **a** Gene structure of the mouse *Bdnf* gene. **b***Bdnf* exon-specific mRNA expression levels in control and CUS groups. *p < 0.05, **p < 0.01 compared with control group.

**Additional file 2 : Supplementary Fig. 2.** Selective deletion of *Bdnf* in the mPFC cannot induce depression-related behaviors in virgin female mice. **a** Schematic of the experimental timeline. **b** Sucrose preference test. **c** Forced swimming test. **d** Novelty-suppressed food test.

## Data Availability

The datasets supporting the conclusion of this article are included within article.

## Abbreviations

AAV: Adeno-associated virus
BDNF: Brain-derived neurotrophic factor
CUS: Chronic unpredictable stress
FST: Forced swim test
FUST: Female urine sniffing test
MDD: Major depressive disorder
mPFC: Medial prefrontal cortex
NSFT: Novelty-suppressed feeding test
PFC: Prefrontal cortex
PPD: Postpartum depression
SCUS: Subthreshold chronic unpredictable stress
SPT: Sucrose preference test
CaMKII: Ca2+/calmodulin-dependent protein kinase
PLC: Phospholipase
PI3K: Phosphatidylinositide 3-kinases
Akt: Protein kinase B
MEK: Mitogen-activated protein kinase
ERK: Extracellular signal-regulated kinase
CREB: cAMP-response element binding protein
NF-ΚB: Nuclear transcription factor-κB
