# Supplementary figures and images for: The BDNF-FoxO1 Axis in the medial prefrontal cortex modulates depressive-like behaviors induced by chronic unpredictable stress in postpartum female mice
Source: Mol Brain. 2020 Jun 12;13:91. doi: 10.1186/s13041-020-00631-3 (PMC7291536; doi:10.1186/s13041-020-00631-3)

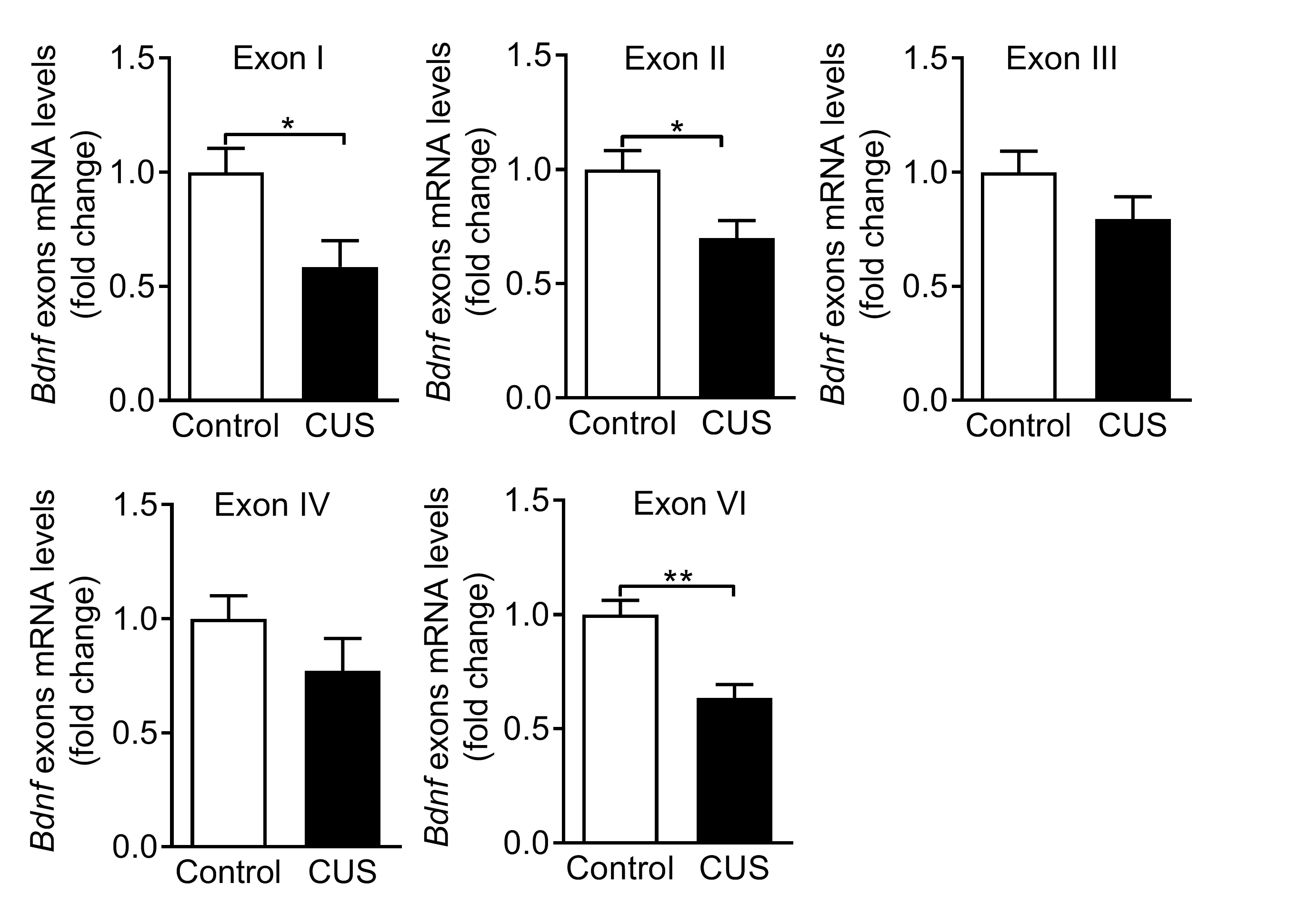

Supplement: Supplementary file 1 — Additional file 1 : Supplementary Fig. 1. Regulation of Bdnf exon mRNA expression in the mPFC by chronic unpredictable stress. a Gene structure of the mouse Bdnf gene. bBdnf exon-specific mRNA expression levels in control and CUS groups. *p < 0.05, **p < 0.01 compared with control group. [file 13041_2020_631_MOESM1_ESM.tif]

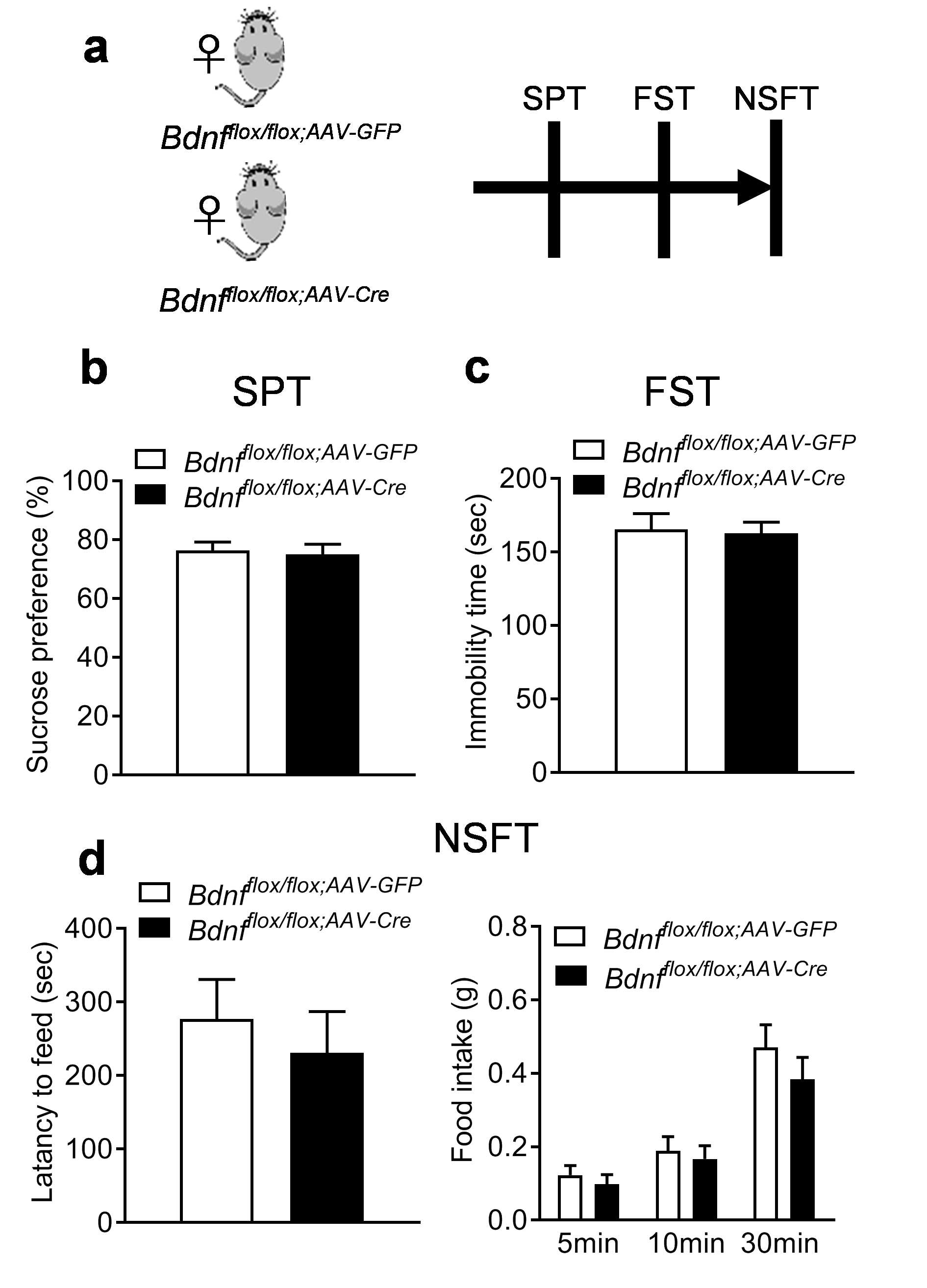

Supplement: Supplementary file 2 — Additional file 2 : Supplementary Fig. 2. Selective deletion of Bdnf in the mPFC cannot induce depression-related behaviors in virgin female mice. a Schematic of the experimental timeline. b Sucrose preference test. c Forced swimming test. d Novelty-suppressed food test. [file 13041_2020_631_MOESM2_ESM.tif]
